# DNA Methylation of HOXA11 Gene as Prognostic Molecular Marker in Human Gastric Adenocarcinoma

**DOI:** 10.3390/diagnostics12071686

**Published:** 2022-07-11

**Authors:** Povilas Ignatavicius, Albertas Dauksa, Justas Zilinskas, Mintaute Kazokaite, Romualdas Riauka, Giedrius Barauskas

**Affiliations:** 1 Department of Surgery, Medical Academy, Lithuanian University of Health Sciences, 50161 Kaunas, Lithuania; albertas.dauksa@lsmuni.lt (A.D.); justas.zilinskas@lsmuni.lt (J.Z.); romualdas.riauka@lsmuni.lt (R.R.); giedrius.barauskas@lsmuni.lt (G.B.); 2 Institute of Digestive Research, Medical Academy, Lithuanian University of Health Sciences, 50161 Kaunas, Lithuania; 3 Institute of Endocrinology, Medical Academy, Lithuanian University of Health Sciences, 50161 Kaunas, Lithuania; mintaute.kazokaite@lsmuni.lt

**Keywords:** gastric adenocarcinoma, methylation, HOXA11, survival

## Abstract

Hypermethylation of tumor suppressor genes and hypomethylation of oncogenes might be identified as possible biomarkers in gastric cancer (GC). We aimed to assess the DNA methylation status of selected genes in GC tissue samples and evaluate these genes’ prognostic importance on patient survival. Patients (99) diagnosed with GC and who underwent gastrectomy were included. We selected a group of genes (RAD51B, GFRA3, AKR7A3, HOXA11, TUSC3, FLI1, SEZ6L, GLDC, NDRG) which may be considered as potential tumor suppressor genes and oncogenes. Methylation of the HOXA11 gene promoter was significantly more frequent in GC tumor tissue (*p* = 0.006) than in healthy gastric mucosa. The probability of surviving longer (71.2 months (95% CI 57–85.3) vs. 44.3 months (95% CI 34.8–53.9)) was observed with unmethylated HOXA11 promoter in cancer tissues. Survival in patients with a methylation of HOXA11 promoter either in healthy gastric mucosa or gastric cancer tissue was twice as high as in patients with a methylation of HOXA11 promoter in both healthy gastric mucosa and cancer tissue (61.2 months (95% CI 50.9–71.4) vs. 28.5 months (95% CI 20.8–36.2)). Multivariate Cox analysis revealed the HOXA11 methylation as significantly associated with patients’ survival (HR = 2.4, 95% CI 1.19–4.86). Our results suggest that the HOXA11 gene might be a potential prognostic molecular marker in patients with gastric adenocarcinoma.

## 1. Introduction

Gastric cancer is one of the most often diagnosed cancers worldwide, with over 1,000,000 new cases annually. It is the fifth most frequently diagnosed malignancy and the third leading cause of cancer mortality (783,000 deaths in 2018) [1,2]. The 5-year survival rate varies from ~10% to 30% in Europe and up to 90% in Japan [1]. A better understanding of the disease development and progression, better early identification and adequate treatment are responsible for decreasing incidence and mortality [3]. However, the global burden of gastric cancer remains high. Several risk factors, such as environmental factors, Helicobacter pylori infection, smoking, and diet are identified [4,5]. Nonetheless, important in the carcinogenesis of gastric cancer are genetic (activation of βcatenin and K-ras, p53 mutations etc.) and epigenetic factors (Deoxyribonucleic acid (DNA) methylation, histone acetylation, chromatin remodeling) [4,6]. In more than 90% of cases in patients with gastric tumors, gastric adenocarcinoma is diagnosed, and two main histological types are identified. Diffuse and intestinal gastric adenocarcinoma differs not only in risk factors, but also in the steps of the carcinogenesis [7,8]. Histological classification of gastric adenocarcinoma has been shown to be an independent prognostic risk factor [7]. When comparing both types, the diffuse gastric adenocarcinoma is more aggressive and worsens the prognosis [9]. Previous studies have shown that location of the gastric adenocarcinoma is also important for the prognosis of the disease. Tumors located in the proximal part of the stomach have worse prognosis, especially when T stage is higher [9,10]. Despite modern treatment modalities such as minimally invasive and cytoreductive surgery, chemotherapy (conventional and hyperthermic intraperitoneal chemoperfusion) in part of the patients with gastric adenocarcinoma diseases recurrence is observed, and the main cause of death is systemic spreading [11]. Today, genetic and epigenetic factors are seen as very important prognostic biomarkers in various cancers. One of the most important epigenetic mechanisms in the carcinogenesis is DNA methylation. It is known that DNA methylation might be induced by various environmental factors, among them smoking, older age, chronic inflammation (reflux esophagitis, ulcerative colitis, chronic hepatitis etc.), and *H. pylori* infection. Therefore, the DNA methylation might be one of the pathways for how all these risk factors are associated with carcinogenesis. During the process of DNA methylation, a methyl group (CH3) is added to the 5′ position of cytosine. Specific regions with high density of CpG are called CpG islands and are in the region of gene promotors. Methylation of these regions is responsible for the changes of gene expression [12]. Hypermethylation of tumor suppressor genes and hypomethylation of oncogenes are two main processes of DNA methylation. However, several other mechanisms involved in these processes are also identified, DNA hypermethylation is not always associated with gene silencing, and DNA hypomethylation does not always mean gene expression. In B cell lymphomas, it was shown that DNA methylation might prevent CTCF-mediated silencing of the oncogene BCL6, and BCL6 transcription was upregulated [13]. In another study, authors observed that expression of tumor suppressor HIC1 in the lymphoid cells was not due to promoter hypermethylation, but some other mechanisms were involved [14]. Multiple studies have shown the importance of these changes as possible biomarkers for the early diagnosis, treatment effectiveness, and disease progression in various cancers [6]. Effective early prediction of the postoperative course of the disease, based on primary tumor tissue DNA methylation changes, might improve the selection and individualization of the treatment in these patients. Therefore, we have chosen a group of several novel epigenetic biomarkers (RAD51B, GFRA3, AKR7A3, HOXA11, TUSC3, FLI1, SEZ6L, GLDC, NDRG) and validated in the set of tissue samples of primary gastric adenocarcinoma, both diffuse and intestinal histological type. The aim of this study was to assess the DNA methylation status of selected genes in gastric cancer tissues samples. Additionally, we evaluated the prognostic importance of the selected gene promoter methylation level for early prediction of gastric cancer progression.

## 2. Materials and Methods

Ninety-nine (99) adult patients were included in the study. All patients were diagnosed with gastric adenocarcinoma and treated at the Department of Surgery, Hospital of Lithuanian University of Health Sciences Kauno Klinikos, Lithuania between January 2010 and December 2017. Diagnosis of gastric adenocarcinoma was based on the final histopathological report. The clinical characteristics of the patients and pathological characteristics of the tumors are summarized in Table 1.

Patients had neither been submitted to chemotherapy or radiotherapy prior to surgery, nor did they have any other diagnosed cancer. The study was approved by the Lithuanian Bioethics Committee (reference number BE-2-17) and written informed consents were obtained from all patients.

### 2.1. Gene Selection

We selected potential tumor suppressor genes (AKR7A3, HOXA11, TUSC3, FLI1, SEZ6L, GLDC, NDRG) and potential oncogenes (RAD51B, GFRA3) of different metabolic pathways, as certain genes, which are involved in cellular pathways such as signal transduction, apoptosis, cell to cell communication, cell cycles and cytokine signaling, are downregulated in cancer.

### 2.2. DNA Samples

Gastric cancer tissues and corresponding healthy gastric mucosa were snap-frozen and stored at −80 °C in liquid nitrogen before DNA extraction. Corresponding healthy gastric mucosa was removed at least 5 mm away from primary tumor. Afterwards, an experienced pathologist reviewed tissue samples of gastric cancer tissues which consisted of at least 80% of cancer cells. Cancer cells were absent in healthy gastric mucosa.

### 2.3. DNA and RNA Extraction, Bisulfite Treatment

Genomic DNA was extracted from 25 to 40 mg of frozen tissues using “AllPrep DNA/RNA Kit” (Qiagen, Hildigen, Germany) according to the manufacturer’s recommendation. DNA concentration was measured using NanoDrop1000 (Thermo Scientific, Wilmington, NC, USA). The methylation status of the gene promoter was determined by bisulfite treatment of DNA. Four hundred (400) ng of DNA was used for bisulfite modification using “EZ DNA Methylation Kit” (Zymo Research, Irvine, CA, USA) according to the manufacturer protocol. Bisulfite treated DNA was eluted and stored in −80 °C until analysis. For a negative and positive methylation control, “Human Methylated & Non-methylated DNA Set” (Zymo research corp, Orange, CA, USA) was used. Promoter methylation was detected by a methylation-specific polymerase chain reaction (MSP). Each MSP incorporated approximately 20 ng of bisulfite treated DNA as a template. Specific primers for methylated and unmethylated DNA sequence were designed using “MethPrimer” [15]. All primers are listed in Table 2.

### 2.4. Methylation-Specific Polymerase Chain Reaction

MSP reaction was performed in 25 μL of total volume, using 10X PCR Buffer (Invitrogen, Waltham, MA, USA), 2 mM MgCl_2_, 0.2 mM dNTP mix (Applied Biosystems, Wilmington, DE, USA) 8% KB Extender, 1.5 U DNA Platinum^®^ Taq DNA Polymerase (Invitrogen, USA), 0.4 µM forward and reverse primers, 20 ng DNA and water. MSP conditions were as follows: initial denaturation at 94 °C for 2 min, followed by 35 cycles of denaturation at 94 °C for 45 s, annealing at 58–62 °C (appropriate for an individual gene) for 90 s, and elongation at 72 °C for 1 min and final extension at 72 °C for 5 min. Amplification products were loaded on a 2% agarose gel with ethidium bromide, and after electrophoresis, gels were documented under Ultraviolet light (UV) using a Gel Doc XR Imaging System (Biorad., Hercules, CA, USA). 

### 2.5. Quantitative Real-Time Polymerase Chain Reaction

The expression levels of HOXA11 were measured with a quantitative reverse transcription polymerase chain reaction (qRT-PCR). First complementary DNA (cDNA) was generated from 500 ng of RNA using High-Capacity RNA-to-cDNA Kit (Applied Biosystems, USA). Amplification reaction was performed in a thermal cycler (Applied Biosystems, USA) using the following cycling profile: 37 °C for 60 min and 95 °C for 5 min. Real-time fluorescence quantitative PCR was performed using ABI 7500 fast Real-Time PCR system (Applied Biosystems, USA). The amplification of specific RNA was performed in a 20 μL reaction mixture containing 3 μg of cDNA template, 1 × TaqMan™ Universal Master Mix II, no UNG (Applied Biosystem, USA) and 1 × TaqMan Gene Expression Assay 20X. The PCR primer used for detection of HOXA11 was from TaqMan, identification number ID: Hs00194149_m1. For normalization, a GAPDH (Hs02758991_g1) housekeeping gene was used. The reaction conditions were as follows: 50 °C for 2 min, 95 °C for 10 min, followed by 40 cycles of 95 °C for 15 s, 60 °C for 60 s. Relative quantification was calculated using the 2^−∆∆Ct^ method.

### 2.6. Statistical Analysis

Descriptive analyses were performed on age, gender, and gastric cancer patient’s clinical characteristics. Differences in methylation levels between cases and controls were analyzed by chi-square test. The Kaplan–Meier survival curves were plotted, and differences in survival between groups of patients were compared using a log-rank test. The Cox regression method was used for univariate and multivariate analysis of variables. All comparisons were considered statistically significant at a *p* < 0.05. Data were analyzed using the SPSS statistical software, version 17.0.0 (SPSS Inc., Chicago, IL, USA).

## 3. Results

**Figure 1:**
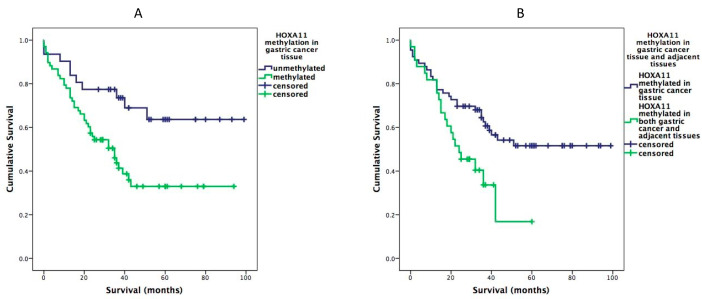
(**A**) Kaplan–Meier analysis showing cumulative survival in gastric cancer patients according to HOXA11 promoter methylation in tumor tissue (log rank = 0.008); (**B**) Kaplan–Meier analysis showing cumulative survival in patients according to HOXA11 promoter methylation in both healthy and tumor tissue vs. methylation in gastric cancer tissue alone (log rank = 0.012).

Methylation analysis was performed in promoter regions of nine selected genes (Table 3). The methylation of the HOXA11 gene promoter was significantly more frequent in gastric cancer tumor tissue (*p* = 0.006) than in healthy gastric mucosa. Promoter region methylation of SEZ6L gene was also clearly higher in gastric cancer tissue than in healthy gastric mucosa (50.5% vs. 29.3%)—however without statistical significance. Methylation (5.1% to 30.3%) of other included genes was also observed. However, there was no difference between methylation in healthy gastric mucosa and gastric cancer tissue. No significant differences were identified when analyzing clinical and morphological characteristics (Table 1) of patients and tumors with methylation of selected genes. The effect of gene methylation on patient survival was evaluated using a Kaplan–Meier log-rank test. The median survival of gastric cancer patients was 54.4 months (95% CI 45.8–62.9). The probability of surviving longer was observed with unmethylated HOXA11 promoter in cancer tissues (71.2 months (95% CI 57–85.3) vs. 44.3 months (95% CI 34.8–53.9) in methylated cases, log rank = 0.008) (Figure 1). Moreover, survival in patients with a methylation of HOXA11 promoter either in healthy gastric mucosa or gastric cancer tissue was twice as high as in patients with a methylation of HOXA11 promoter in both healthy gastric mucosa and cancer tissue (61.2 months (95% CI 50.9–71.4) vs. 28.5 months (95% CI 20.8–36.2)) (Figure 1). In a univariate Cox analysis, we identified tumor size (pT) (HR = 5.95, 95% CI 1.8–19.13), lymph nodes metastasis (HR = 3.59, 95% CI 1.6–7.99), invasion into blood vessels (HR = 2.65, 95%PI 1.35–5.2), invasion into lymph vessels (HR = 3.8, 95% CI 1.37–10.6) and HOXA11 promoter methylation (HR = 2.48, 95%PI 1.24–5) as significantly associated with shorter survival. Only a methylation HOXA11 gene promoter was identified as significantly associated with patient’s survival in multivariate Cox analysis (HR = 2.4, 95% CI 1.19–4.86) (Table 4).

**Figure 2:**
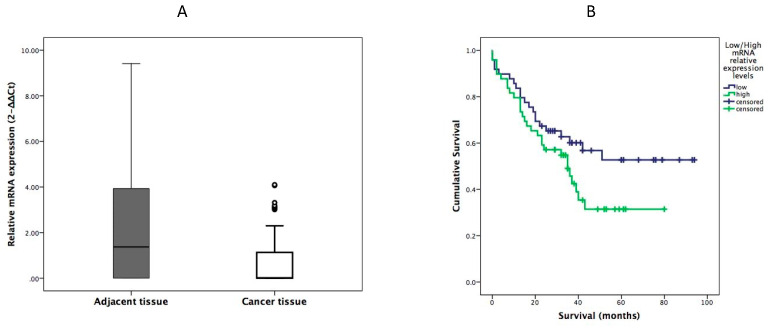
(**A**) Comparison of the relative expression of HOXA11 in gastric cancer tissue and adjacent healthy tissue. Data presented as mean ± SD; (**B**) Kaplan–Meier analysis showing cumulative survival in patients according to HOXA11 mRNA expression levels (low vs. high) in cancer tissue.

We assessed the mRNA expression of hypermethylated gene HOXA11 in gastric cancer tissue and adjacent healthy mucosa by using RT-qPCR. The relative mRNA expression of HOXA11 was significantly higher in adjacent healthy gastric mucosa than that in gastric cancer tissue. mRNA expression in gastric cancer tissue was downregulated by 1.9-fold (*p* = 0.002) (Figure 2).

We did not find any association between HOXA11 mRNA expression and histopathological tumor features. However, we found that mRNA expression levels are associated with a patient’s survival. Patients with mRNA expression below the median values in cancer tissues were assigned as having low expression levels, and patients with mRNA above or equal to median assigned as having high expression levels. Patients with high mRNA expression levels had shorter survival than patients with low mRNA expression levels (39.6 months (95% CI 30.5–48.7 vs. 58.8 months (95% CI 47.4–70.2)) (Figure 2).

**Figure 3:**
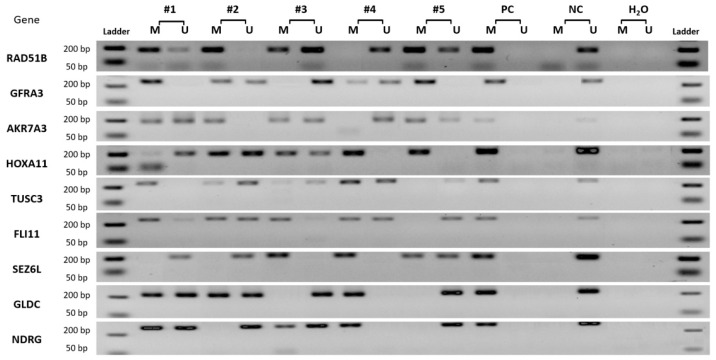
Representative gel electrophoresis pictures of MS-PCR products demonstrating aberrant methylation of selected genes in gastric cancer patients. M and U, the methylated and unmethylated primers, respectively. For a positive (PC) and negative (NC) methylation control, “Human Methylated & Non-methylated DNA Set” was used. Water was used as a negative control for each amplification. The presence of a correct molecular weight PCR product signal indicates the presence of either unmethylated or methylated alleles.

Representative gel electrophoresis pictures of MS-PCR products demonstrating aberrant methylation of selected genes in gastric cancer patients are shown in Figure 3.

## 4. Discussion

In the present study, we investigated the methylation status of selected genes in a tumor and adjacent healthy tissue in patients with gastric adenocarcinoma. Selected genes might be considered as potential tumor suppressor genes (AKR7A3, HOXA11, TUSC3, FLI1, SEZ6L, GLDC, NDRG) and oncogenes (RAD51B, GFRA3). The analysis showed that the HOXA11 gene promoter was significantly more frequently methylated in cancer tissue than in healthy adjacent tissue (68.7% vs. 39.4%). Patients in which HOXA11 promoter in cancer tissue was methylated survived for a shorter time (44.3 months vs. 71.2 months). Moreover, if HOXA11 promoter methylation was detected in both healthy and tumor tissue, overall survival was only 28.5 months. Methylation of HOXA11 promoter was also significantly associated with shorter survival in univariate and multivariate Cox analysis. These findings are important in demonstrating that methylation of the HOXA11 gene might be a good prognostic molecular marker in patients with gastric adenocarcinoma, especially because this disease is a cause of almost 800,000 yearly deaths worldwide [2]. HOXA11 (Homeobox A11) is a Protein Coding gene and a member of HOX transcription factors. Genes of the HOX family are an important part of region-specific development of the vertebrate body plan [16]. HOXA11 has several functions also in the human body. It is involved in the development of endometrium and appears to be regulated by ovarian steroids [17]. In the last few years, more and more studies show that HOXA11, through various mechanisms, is involved in the development and progression of various cancers. HOXA11 DNA methylation has already been identified as a potential biomarker in several cancers such as renal cell carcinoma, ovarian and cervical cancer, endometrial adenocarcinoma and non-small cell lung cancer [18,19,20]. HOXA11 is also a known gene in gastrointestinal (GI) cancers. However, the number of studies is low and different mechanisms are analyzed. There are even fewer studies analyzing methylation patterns of HOXA11 in GI cancers. Several overexpression and knockdown studies have shown that HOXA11 is involved in regulating the liver metastasis in colorectal cancer cell lines [21]. In some previous studies, several pathways of HOXA11 functions in the development and progression of hepatocellular carcinoma (HCC) were analyzed. In a current study, Liu et al. demonstrated that HOXA11-AS functions even as an oncogene promoting the progression of HCC [22]. Another recent study suggested that silencing the HOXA11-AS activity via a Wnt signaling pathway might have an antitumor effect and suppress the development of HCC by decreasing the methylation level of the promoter region of HOXA11 [23]. Only a few studies have investigated the role of HOXA11 in the carcinogenesis and progression of gastric adenocarcinoma. Therefore, the results of the present study are important and timely. In a recent study, after measuring levels of the HOXA11-AS in the gastric cancer tissues, cell line and serum samples, the role of HOXA11-AS in the diagnosis and prognosis was evaluated. The authors concluded that HOXA11-AS might be a potential biomarker by promoting cell proliferation and invasion of gastric cancer [24]. However, HOXA11-AS is involved not only in the promotion of cell proliferation and invasion but also in promotion of gastric cancer cell metastasis [25]. Already in 2014, Bai et al. in a small study sample (32 gastric cancer samples) showed that methylation of HOXA11 is higher in gastric cancer tissue and adjacent tissues than compared to normal gastric mucosa from healthy participants. However, in comparison to our study, no survival analysis was performed, and the clinical relevance of findings was not totally clear at that time [26]. Cui et al. performed a larger analysis with seven gastric cancer cell lines, five cases of normal gastric mucosa and 112 cases of gastric cancer tissue. They showed that HOXA11 is associated with gastric cancer proliferation, migration, and invasion. However, the survival analysis was not performed and therefore the clinical significance is not clear [27]. This study contains several limitations. First, epigenetic changes of only one gene from a selected nine were identified as promising molecular biomarkers in gastric cancer. This might be influenced by selection bias, as for analysis we selected only genes which were identified as potential biomarkers in other cancers and data on gastric cancer were missing. Secondly, due to complexity and cost of the epigenetic tissue analysis, the results of this study are still applicable mostly as part of clinical studies.

## 5. Conclusions

DNA methylation of HOXA11 gene promoter is more frequent in gastric cancer tumor tissue than in healthy gastric mucosa and is associated with shorter postoperative survival in patients diagnosed with gastric adenocarcinoma. Our results suggest that the HOXA11 gene might be a prognostic molecular marker in patients with gastric adenocarcinoma.

## Figures and Tables

**Table 1:** Clinical and pathological characteristics.

		n (%)
Age	<60	25 (25.3)
	≥60	74 (74.7)
Gender	Female	34 (34.3)
	Male	65 (65.6)
pT category	I/II	22 (22.2)
	III/IV	77 (77.8)
Lymph node metastasis (N)	Negative	30 (30.3)
	Positive	69 (69.7)
Distant metastasis (M)	Negative	80 (80.8)
	Positive	19 (19.2)
Invasion into blood vessels (V)	Negative	30 (30.3)
	Positive	58 (58.6)
Invasion into lymph vessels (L)	Negative	18 (18.2)
	Positive	71 (71.7)
Tumor grade	G1/G2	32 (32.3)
	G3	59 (59.6)
HER receptors	Negative	62 (62.6)
	Positive	18 (18.2)
Histological type	Intestinal	43 (43.4)
	Diffuse/mixed	49 (49.5)

**Table 2:** Methylation-specific polymerase chain reaction primers.

Gene	Forward Primer 5′-3′	Reverse Primer 5′-3′	T
RAD51B-M	TAGGTTTAAGTGATTTTTTCGTTTC	ATAATCTCGATTTCCTAACCTCGTA	61
RAD51B-U	AGGTTTAAGTGATTTTTTTGTTTTG	ATAATCTCAATTTCCTAACCTCATA	
GFRA3-M	ATGTTGTTGTTGTTGTTGTCGTC	AACACTCAATCTATCTCAATTCGTA	59
GFRA3-U	ATGTTGTTGTTGTTGTTGTTGTTG	AACACTCAATCTATCTCAATTCATA	
AKR7A3-M	TTGAGTAGTTGAGATTATAGGGGTAC	AAAAAACTAAAAACGATAATTCACG	60
AKR7A3-U	AGTAGTTGAGATTATAGGGGTATGT	AAAACTAAAAACAATAATTCACACC	
HOXA11-M	AGTACGTATAAAGGTAGCGTTTTGC	CCTTAACCGATAAATATTTCACGAC	57
HOXA11-U	TATGTATAAAGGTAGTGTTTTGTGT	CCTTAACCAATAAATATTTCACAAC	
TUSC3-M	TCGAAGTTTGGTTTTTTCGTTAC	GACAAAACAATATCTCCTCCACG	59
TUSC3-U	TTGAAGTTTGGTTTTTTTGTTATGT	AACAAAACAATATCTCCTCCACAC	
FLI1-M	GAAGGAAATAACGAATAATTTTGTC	AATTAACTTCTATTTCTCAAACGTT	58
FLI1-U	AGGAAATAATGAATAATTTTGTTGT	AATTAACTTCTATTTCTCAAACATT	
SEZ6L-M	GTTTAAAATCGGGGTTAGGAATC	GTTTAAAATCGGGGTTAGGAATC	55
SEZ6L-U	GTTTAAAATTGGGGTTAGGAATTGT	CAAAAATTTAAAATTTAAAAACAAC	
GLDC-M	GTTTACGTTTGTAATGACGGATTAC	CCTAACTAAAATACAATAACGCGAT	57
GLDC-U	TTATGTTTGTAATGATGGATTATGA	CCCTAACTAAAATACAATAACACAAT	
NDRG-M	GGAATTTAGGGAGGAGTAGAGTTTC	AATTCACCTCCATTATCTAAACGAA	58
NDRG-U	GGAATTTAGGGAGGAGTAGAGTTTT	AATTCACCTCCATTATCTAAACAAA	

M: methylated; U: unmethylated; T: annealing temperature.

**Table 3:** Methylation frequency in healthy gastric mucosa and cancer tissue.

Gene	Healthy Gastric Mucosa		Gastric Cancer Tissue		*p*
	Methylated Cases/ All Cases (n)	Methylation Frequency (%)	Methylated Cases/ All Cases (n)	Methylation Frequency (%)	
RAD51B	5/99	5.1	7/99	7.1	0.247
GFRA3	24/99	24.2	30/99	30.3	0.378
AKR7A3	17/99	17.2	22/99	22.2	0.154
HOXA11	39/99	39.4	68/99	68.7	0.006
TUSC3	22/93	22.2	21/93	21.2	0.491
FLI1	9/96	4	8/96	5.1	0.113
SEZ6L	29/99	29.3	50/99	50.5	0.054
GLDC	28/88	31.8	26/88	29.5	0.365
NDRG	3/91	3.3	6/91	6.6	0.64

**Table 4:** Survival analysis.

Univariate COX Regression Analysis			
	HR	95% CI	*p*
Age	1.02	0.99–1.05	0.13
Gender	1.6	0.9–3.3	0.12
Tumor size (pT)	5.95	1.8–19.13	0.003
Lymph node metastasis (N)	3.59	1.6–7.99	0.002
Distant metastasis (M)	1.85	0.97–3.55	0.064
Invasion into blood vessels (V)	2.65	1.35–5.2	0.005
Invassion into lymph vessels (L)	3.8	1.37–10.6	0.01
Tumor grade	1.01	0.55–1.87	0.97
HER receptor positivity	0.98	0.46–2.06	0.96
Histological type	1	0.55–1.8	0.99
HOXA11 methylation	2.48	1.24–5	0.011
SEZ6L methylation	1.29	0.74–2.27	0.36
Multivariate COX regression analysis			
Tumor size (pT)	3.2	0.9–11.4	0.07
Lymph node metastasis (N)	2.12	0.8–5.6	0.13
Invasion into blood vessels (V)	1.27	0.32–4.67	0.77
Invasion into lymph vessels (L)	1.19	0.55–2.58	0.66
HOXA11 methylation	2.4	1.19–4.86	0.015

HR: hazard ratio; CI: confidence interval.

## Data Availability

The datasets used and analyzed during the current study are available from the corresponding author on reasonable request.
